# High Entrainment Constrains Synaptic Depression Levels of an *In vivo* Globular Bushy Cell Model

**DOI:** 10.3389/fncom.2017.00016

**Published:** 2017-03-20

**Authors:** Marek Rudnicki, Werner Hemmert

**Affiliations:** Bio-Inspired Information Processing, Technische Universität MünchenMünchen, Germany

**Keywords:** globular bushy cell, cochlear nucleus, short term depression, auditory pathway, high-sync neurons, temporal processing, sound localization, computational model

## Abstract

Globular bushy cells (GBCs) located in the ventral cochlear nucleus are an essential part of the sound localization pathway in the mammalian auditory system. They receive inputs directly from the auditory nerve and are particularly sensitive to temporal cues due to their synaptic and membrane specializations. GBCs act as coincidence detectors for incoming spikes through large synapses—endbulbs of Held—which connect to their soma. Since endbulbs of Held are an integral part of the auditory information conveying and processing pathway, they were extensively studied. Virtually all *in vitro* studies showed large synaptic depression, but on the other hand a few *in vivo* studies showed relatively small depression. It is also still not well understood how synaptic properties functionally influence firing properties of GBCs. Here we show how different levels of synaptic depression shape firing properties of GBCs in *in vivo*-like conditions using computer simulations. We analyzed how an interplay of synaptic depression (0–70%) and the number of auditory nerve fiber inputs (10–70) contributes to the variability of the experimental data from previous studies. We predict that the majority of synapses of GBCs with high characteristic frequencies (CF > 500 Hz) have a rate dependent depression of less than 20%. GBCs with lower CF (<500 Hz) work also with strong depressing synapses (up to 50% or more). We also showed that synapses explicitly fitted to *in vitro* experiments with paired-pulse stimuli did not operate properly in *in vivo*-like conditions and required further extension to capture the differences between *in vitro* and *in vivo* experimental conditions. Overall, this study helps to understand how synaptic properties shape temporal processing in the auditory system. It also integrates, compares, and reconciles results of various experimental studies.

## 1. Introduction

Globular bushy cells (GBCs) are located in the ventral cochlear nucleus, which is the first processing station in the central auditory nervous system. Cochlear nucleus receives direct inputs from the inner ear through auditory nerve fibers (ANFs). In addition to GBCs, the population of neurons in ventral cochlear nucleus is made of several different types: spherical bushy cells, stellate cells, and octopus cells (Osen, 1969; Rhode et al., 1983). Each cell type processes different sound features, e.g., spectrum, temporal fine structure, or signal onset (Recio, 2000). The pre-processing of sound by the cochlear nucleus neurons is essential for higher auditory centers to perform their functions, e.g., sound localization or sound identification.

GBCs enhance temporal sound cues which are essential for sound localization. When stimulated with pure tones, their action potentials lock precisely to a certain phase of the signal. Some of the GBCs are experts in phase-locking and reach synchronization levels higher than any ANF. Neurons with such properties are sometimes called “high-sync” neurons (Joris et al., 1994). Additionally, they can fire an action potential every stimulus cycle up to 700 Hz, which is more than twice the maximum firing rate for ANFs. GBCs similarly show increased synchronization and entrainment in response to complex sounds (Rhode and Greenberg, 1994).

Rothman et al. (1993) explained the mechanism of high synchronization (temporal precision of spikes) and high entrainment (one spike for each stimulus cycle) by the presence of many converging inputs. GBCs receive their main excitatory inputs from ANFs directly onto the soma through large synapses called modified endbulbs of Held (Spirou et al., 2005). To elicit an action potential, multiple subthreshold ANF input spikes must coincide. As a result, GBC spikes have greater temporal precision than the input action potentials from individual ANFs.

In most *in vitro* experiments, the strength of the excitatory inputs (excitatory postsynaptic currents, EPSC, synaptic strength) is strongly reduced after a presynaptic spike as shown in **Figure 3**. In the time between two input spikes, the synaptic strength recovers and if the pause between stimulations is long enough, it eventually reaches its original value (Wang and Manis, 2008; Yang and Xu-Friedman, 2009, 2015). This synaptic depression at the endbulb of Held is associated mostly with the depletion of neurotransmitter at the presynaptic site (Friauf et al., 2015) and receptor desensitization (Yang and Xu-Friedman, 2008). The level of depression depends strongly on the stimulation frequency (Yang and Xu-Friedman, 2009). A similar behavious was observed in a similar auditory synapse in medial nucleus of the trapezoid body, calyx of Held (Taschenberger et al., 2016). In contrast to *in vitro* experiments, *in vivo* extracellular recordings do not indicate strong variations of synaptic strength at the endbulbs of Held during stimulation (Young and Sachs, 2008; Borst, 2010; Kuenzel et al., 2011, 2015). The few *in vivo* studies of endbulb of Held can be complemented with *in vivo* studies of calyx of Held, that shows slight decrease in synaptic strength only at high stimulation frequencies (Lorteije et al., 2009; Di Guilmi et al., 2014).

Currently, the difference between the *in vitro* and *in vivo* observations is not well understood. In particular, how the different levels of depression influence the firing properties in response to sounds is unclear. Both endbulb and calyx of Held are regarded as “model synapses.” It is therefore important to obtain a precise understanding of their operation. In this study, we present a biophysically plausible computational model of GBCs, which is driven by realistic *in vivo*-like stimuli. The main goals of the study were:

- To optimize the synaptic parameters of endbulbs of Held to accurately simulate GBCs including “high-sync” neurons (Sections 2.1 and 2.3).
- To better understand the behavior of synapses with different depression levels (from tonic, through weakly depressing, to strongly depressing as seen in *in vitro* experiments) in *in vivo*-like simulations (**Figure 5**, Sections 3.1 and 3.3).
- To demonstrate how the higher number of ANF inputs increases the firing rate and the entrainment of bushy cells (Section 3.2). Experimental evidence shows similar variability (Joris et al., 1994; Spirou et al., 2005).
- To provide a more comprehensive auditory model by combining an inner ear model with a GBC model, that can be used for further processing, e.g., for sound localization models. In this case, it is not sufficient that both models independently generate realistic outputs but also their combination, which provides further constraints.

## 2. Methods

### 2.1. Model overview

**Figure 1:**
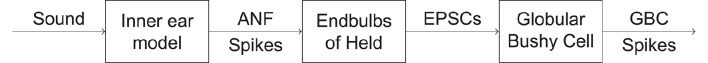
**Structure of the GBC model**. ANFs converge onto a soma of a GBC and excite it through large synapses (endbulbs of Held).

The model consisted of three main components: (1) ANF inputs generated by an inner ear model (Zilany et al., 2014), (2) endbulbs of Held, and (3) a soma of a bushy cell. The general structure is shown in Figure 1 and was derived from the previous work of Rothman et al. (1993).

First, the sound enters the cat inner ear model by Zilany et al. (2014) and is converted to ANF spike trains. The model of Zilany et al. (2014) is a state-of-the-art model capable of generating ANF responses with typical adaptation properties an with realistic phase locking (Zilany et al., 2009). The model includes several processing stages, such as a basilar membrane, inner hair cells, power-law dynamics at the inner hair cell synapses, and spike generators.

Next, we selected a number of ANF spike trains to drive synapses that were attached to the soma of the bushy cell. According to an electron microscopy study by Spirou et al. (2005), the number of ANF inputs shows large variations: between 9 and 69 ANF inputs across a population of 12 bushy cells. We simulated GBCs with the number of inputs varying from 10 to 70 per bushy cell. The inputs were coming solely from high-spontaneous rate fibers. We neglected low and medium spontaneous rates fibers after we verified (not shown) that the inclusion of a small number of them does not have significant influence on the firing properties in the simulated experiments Liberman (1991). The model synapses were instantly activated by ANF input spikes and had an exponential decay of the conductance with a short 0.2 ms time constant (Yang and Xu-Friedman, 2009; Cao and Oertel, 2010). The summation of multiple synaptic conductances was linear. With respect to synaptic depression, we examined three different synapse types: (1) a tonic synapse model, (2) single exponential recovery synapse with different levels of synaptic depression, and (3) double exponential recovery synapse tuned to *in vitro* experiments (Section 2.2).

The final component of the model was the soma of a GBC. We neglected dendritic trees, because the dendritic inputs are not well known and somatic ANF inputs are the major ones (Spirou et al., 2005). Each GBC was modeled as a single compartment with Hodgkin–Huxley-like channels (Rothman and Manis, 2003a,b,c) described by the following equation:

(1) $$\begin{array}{l} I=C\frac{dV}{dt}+I_{leak}+I_{Na}+I_{Kht}+I_{Klt}+I_{h}, \end{array}$$

where *I* is the membrane current, *C* is the membrane capacity, *V* denotes the membrane voltage, *I*_*Klt*_ is the fast-activating slow-inactivating low-threshold *K*^+^ current, *I*_*Kht*_ is the high-threshold *K*^+^ current, *I*_*h*_ is the hyperpolarization-activated cation current, *I*_*leak*_ is the leakage current, and *I*_*Na*_ is the *Na*^+^ current. The model was derived directly from Rothman and Manis (2003c) with the exception of sodium channels taken from Spirou et al. (2005). The original sodium channels produced unrealistically long refractory periods and needed to be replaced by channels with faster dynamics. The substitution resulted in the absolute refractory period of 0.66 ms. All channel parameters are summarized in Table 1 (Model Type II) of Rothman and Manis (2003c) and in the Appendix of Spirou et al. (2005) with the following adjustments. Time constants were corrected for the *in vivo* temperature of 37°C using *Q*_10_ = 2.5 for sodium and *Q*_10_ = 3 for all other channels. To match measured firing thresholds (Yang and Xu-Friedman, 2009), the maximum *Na*^+^ channel conductance was increased to 2,500 nS.

**Table 1:** **Synapse types**.

**Name**	**Description**
Tonic	Constant synaptic weight
X%-depressing	Synapse with a single exponential recovery depressing by *X%* when stimulated at 300 Hz (Equation 3)
yang2009mean	Synapse with a double exponential recovery and mean parameters of an endbulb population from *in vitro* experiments by Yang and Xu-Friedman (2009)

### 2.2. Modeling of synaptic depression

Table 1 lists synapse models used in this study.

The tonic synapse was the first and the simplest model that was examined. The synaptic weight was constant and resulted in constant EPSCs that did not depend on the ANF firing rates. Even though endbulbs of Held might not be perfectly tonic synapses, especially at higher stimulation rates (Young and Sachs, 2008; Borst, 2010), this model is equivalent to a 0%-depressing synapse and defines the bound of the parameter space.

The second model was a depressing synapse with a single exponential recovery (Tsodyks and Markram, 1997) which was referred as X%-depressing synapse throughout this study. The purpose of this model was to examine a wide range of synaptic depressions spanning from strong *in vitro* to weak depression observed in some *in vivo* experiments. The synapses were fitted only in the *in vivo* operational stimulation range. The peak synaptic conductance was proportional to the active internal synaptic resources. Every pre-synaptic spike caused an activation of some synaptic resources. Between the spikes, the synapse could recover exponentially. This process can be expressed iteratively with Equation (2):

(2) $$\begin{array}{l} g_{n+1}=g_{n}\left(1-u\right)e^{-\Delta t/\tau }+w\left(1-e^{-\Delta t/\tau }\right), \end{array}$$

where *w* is the peak conductance of a completely recovered synapse, *u* is the fraction of available synaptic resources activated at each stimulation. As stated by Tsodyks and Markram (1997), depending on the physical mechanisms, *u* can be partially or completely determined by release probability of the neurotransmitter triggered by a presynaptic spike. Δ*t* is the time from the last (*n*-th) synaptic event, and τ is the synaptic recovery time constant. τ was set to 90 ms based on *in vitro* experiments with *in vivo*-like stimuli of endbulbs (Yang and Xu-Friedman, 2015) and calyx of Held (Hermann et al., 2009). The initial value of *g* (*g*_0_) was set to *w*/(1−*u*) so that *g*_1_ had the peak conductance of *w*. The depression level *X%* is defined as a relative decrease of steady-state synaptic strength *s*_*f*_ between the spontaneous rate (SR) (*f* = 50Hz) and the driven rate (*f* = 300Hz). It can be calculated with Equation (3):

(3) $$\begin{array}{l} X\%=\left(1-\frac{s_{300\text{Hz}}}{s_{50\text{Hz}}}\right)\cdot 100. \end{array}$$

**Figure 2:**
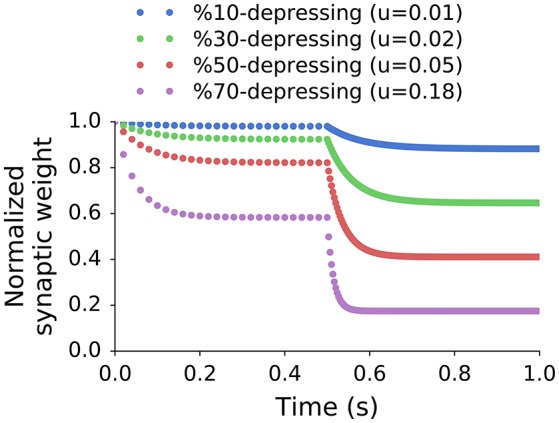
**Normalized peak synaptic weights of single exponential recovery synapses stimulated at two different frequencies**. The stimulation in the first interval (0–0.5 s) was 50 Hz (approximately SR of the input), and 300 Hz (approximately max rate of input) in the second one (0.5–1 s). The steady states in each interval (*s*_*f*_) were used to calculate the effective depression level (*X%*) using Equation (3). All synapses share the same recovery time constant but have different values of *u*.

The value of *X%* can be set by adjusting *u* in the model, because it directly changes the steady-state synaptic weights (*s*_*f*_) as shown in Figure 2. The stimulation frequencies of 50 and 300 Hz were chosen as they correspond approximately to an input operational range bound by the spontaneous and driven rates of ANFs. In other studies, especially *in vitro*, *X%* can have a different meaning and refer to depression relative to an unconditioned synapse. As a result, the depression values are typically much higher in *in vitro* synapses.

**Figure 3:**
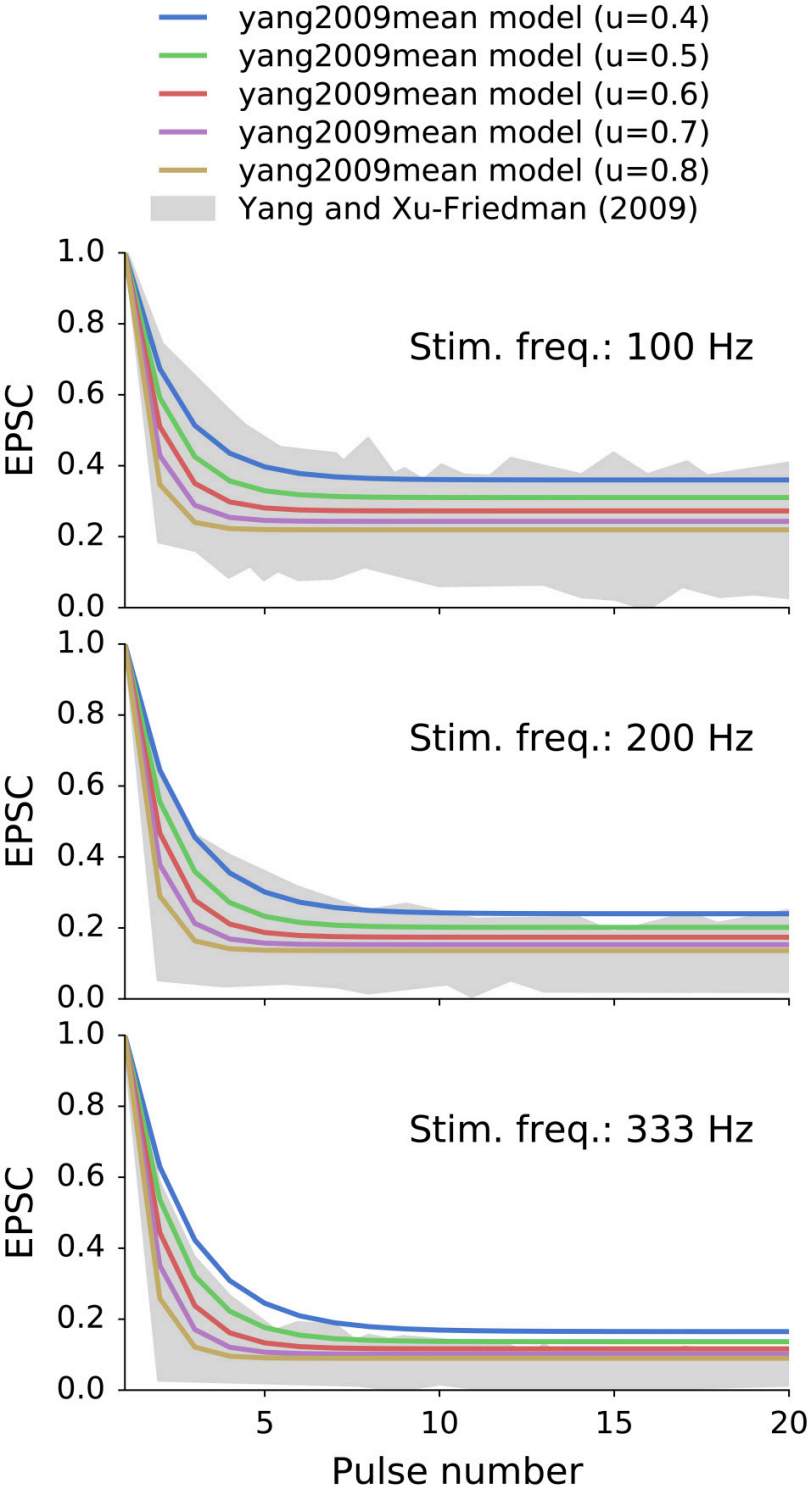
**Synaptic depression of a double exponential recovery synapses (yang2009mean) with different values of *u* that were stimulated at 100, 200, 333 Hz**. Synapse's recovery times constants were equal to the mean population values from *in vitro* experiments of Yang and Xu-Friedman (2009). The selected value of *u* was 0.6 as measured by Oleskevich et al. (2000) *in vitro*. The grayed area represents the population data from Yang and Xu-Friedman (2009).

The third synapse model was a depressing synapse with a double exponential recovery. It is called yang2009mean mean in this study, because it is based on the mean *in vitro* data from Yang and Xu-Friedman (2009). The purpose of this model is to replicate behavior of synapses measured *in vitro* and validate them in simulated *in vivo*-like conditions. In their experiments, Yang and Xu-Friedman (2009) observed two exponential components in the recovery of EPSCs. Thus a model based on double exponential recovery, such as Cook et al. (2003), was a natural match to the experimental data. The model is described by Equation (4) and the parameters were set to the mean values of the population from Yang and Xu-Friedman (2009).

(4) $$\begin{array}{l} \begin{array}{c} g_{n+1}=k\left(g_{n}\left(1-u\right)e^{-\Delta t/\tau_{f}}+w\left(1-e^{-\Delta t/\tau_{f}}\right)\right)+\\ +\;\left(1-k\right)\left(g_{n}\left(1-u\right)e^{-\Delta t/\tau_{s}}+w\left(1-e^{-\Delta t/\tau_{s}}\right)\right), \end{array} \end{array}$$

where *w* is the peak conductance of a completely recovered synapse, *u* is the fraction of the available synaptic resources being utilized at presynaptic event. We interpret this value as the vesicle release probability of 0.6, that was found for endbulbs of Held *in vitro* (Oleskevich et al., 2000). Δ*t* is the time from the last (*n*-th) synaptic event, τ_*s*_ (1,990 ms) and τ_*f*_ (10.9 ms) are the slow and fast synaptic recovery time constants, respectively, and *k* (0.3) is the fraction of the fast recovery (relative to the slow recovery). The initial value of *g* (*g*_0_) was set to *w*/(1−*u*). The model exhibits different depression levels depending on the stimulation frequency. Figure 3 shows normalized EPSC of the model with different values of *u* for reference that was stimulated at 100, 200, and 333 Hz. They grayed area corresponds to the population data obtained in an analogous *in vitro* experiment from Yang and Xu-Friedman (2009).

It is worth to note that Equation (2) describing X%-depressing models can be derived from Equation (4) of the yang2009mean model by setting *k* to 1. Also, the tonic synapse is a special case of X%-depressing model, where *X* = 0. However, because the models have different motivations and were fitted differently, they are presented as three distinct models in this study.

### 2.3. Fitting of synaptic weights

Modeling of GBCs, and “high-sync” neurons in particular, required adjustment of synaptic weights of the endbulbs of Held. The experiments of Joris et al. (1994) were used as a reference for this procedure. In our simulations, bushy cells were simulated with a *in vivo*-like stimulation protocol which consists of several steps:

1. A signal was generated. It consisted of 100 gated tones that were repeated every 100 ms and each tone lasted 25 ms. The frequency of the tones was equal to the CF of the stimulated neuron and their intensity was 50 dB_SPL_.
2. An inner ear model driven by that signal was used to generate the ANF spike trains.
3. The ANF spike trains were used to stimulate models of endbulbs of Held and GBCs.
4. The output spike trains of GBCs were recorded.

After each simulation, vector strength (VS), firing rate, and entrainment index (EI) were calculated from responses of GBCs.

VS is a measure that specifies the strength of phase locking in response to periodic stimuli (usually pure tones). The VS is calculated assuming that each spike represents a unit vector $\hat{v}$ with a phase equal to the phase of the stimulus at which it occurred. VS is equal to the magnitude of the sum of all such vectors normalized by the number of vectors *n*:

(5) $$\begin{array}{l} VS=\frac{\left|\sum \hat{v_{i}}\right|}{n}. \end{array}$$

The values of VS are between 0 and 1. VS of 0 indicates that spikes are uniformly distributed over the stimulus period and 1 indicates that all spikes occur at exactly the same phase of the stimulus. “High-sync” neurons are defined in terms of VS > 0.9 for CF < 700 Hz (Joris et al., 1994).

EI specifies whether a neuron fired action potentials once for every period of the stimulus or less. The entrainment index *p* is calculated using the definition given by Joris et al. (1994). First an inter-spike interval histogram is constructed. Then the number of intervals *k* belonging to the first maximum (between 0.5 and 1.5 of the stimulus period) is divided by the total number of intervals *n* as given by:

(6) $$\begin{array}{l} p=\frac{k}{n}. \end{array}$$

The values of EI are between 0 and 1. A value of 1 indicates that the neuron fired a minimum of one spike during each stimulus period. Entrainment falls to 0 when the neuron can no longer fire action potentials for adjacent stimulus periods.

**Figure 4:**
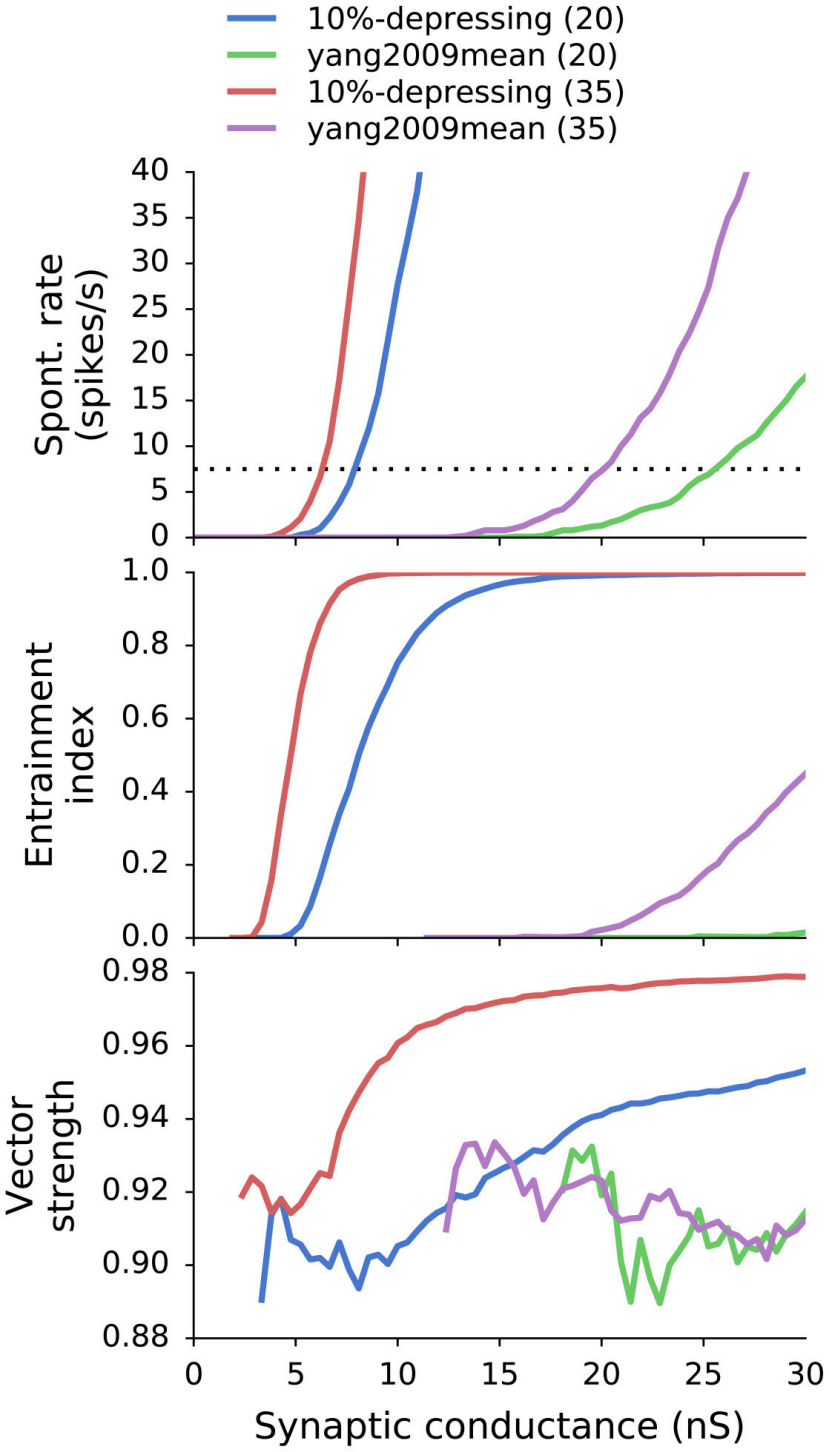
**SR, EI, and VS as a function of synaptic weight for a combination of two synapse models (10%-depressing and yang2009mean) and two different number of ANF inputs (20 and 35)**. SR and EI increase with increasing synaptic weight. VS also increases with synaptic weight for 10%-depressing synapses, but does not increase for synapses tuned to Yang and Xu-Friedman (2009). The thin dotted black line marks the desired SR of 7.5 spikes/s (Spirou et al., 1990).

Generally, VS, EI, and SR were calculated for each combination of synapse type *T* (where *T* ∈ {tonic, X%-depressing, yang2009mean}), ANF number of inputs *N* (where *N* ∈ [10, 70]), and synaptic weight *w*. For simplicity, all synaptic weights had equal values for a given GBC. The parameter scan resulted in the following mapping: (*T, N, w*) → (*VS, EI, SR*). VS, EI, and SR can be seen as functions of *w* for all combinations of *T* and *N*: VS_*T, N*_(*w*), EI_*T, N*_(*w*), SR_*T, N*_(*w*). As an example, results with two synapse types and two different values of *N* are shown Figure 4.

On the one hand, an increase in *w* causes an increase in VS and EI, which is desirable for GBCs. On the other hand, increasing *w* can cause unrealistically high SR. As a result, the optimal weights were found by increasing the value of *w* up to the point where *SR*_*T, N*_(*w*) crossed the mean experimental SR from Spirou et al. (1990) which equals to 7.5 ± 13.8 spikes/s and is valid only for GBCs with CF < 6 kHz. The procedure of finding the best weight *w*_*o*_ is equivalent to Equation (7):

(7) $$\begin{array}{l} w_{o}=\underset{w}{\text{arg min}}|\text{SR}\left(w\right)-{\text{SR}}_{t}|, \end{array}$$

where SR_*t*_ is the target spontaneous rate (7.5 spikes/s). The procedure of finding optimal weights is further discussed in Section 4.1.

**Figure 5:**
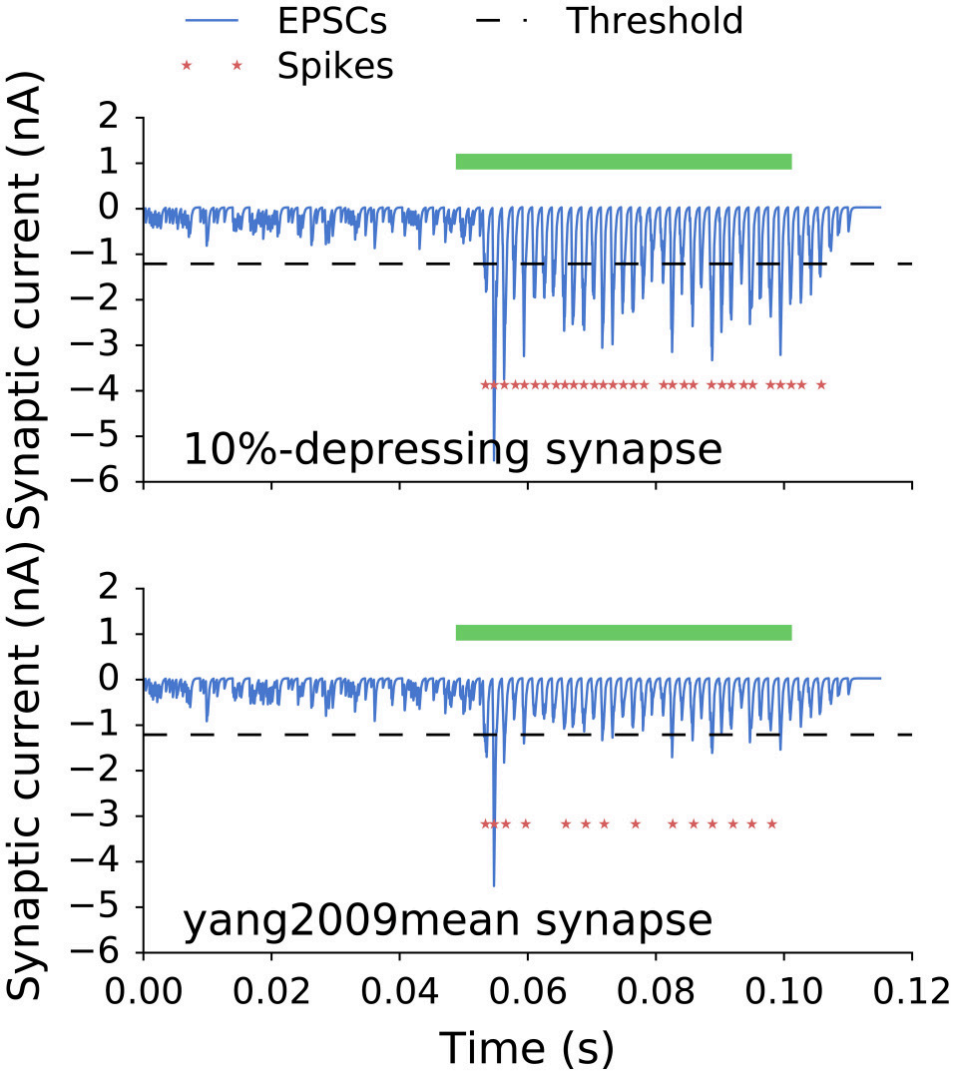
**Postsynaptic currents of two GBCs with 10%-depressing and yang2009mean synapses stimulated with a 50 ms pure tone of 650 Hz (green bar)**. The dashed line is plotted for reference and indicates a threshold for a single EPSC to initiate an action potential. Red stars indicate action potentials triggered by the stimulation. The less depressing synapses (10%-depressing) produced more action potentials than the more depressing synapses (yang2009mean, Table 1). The SR for both cells was 7.5 spikes/s.

**Figure 6:**
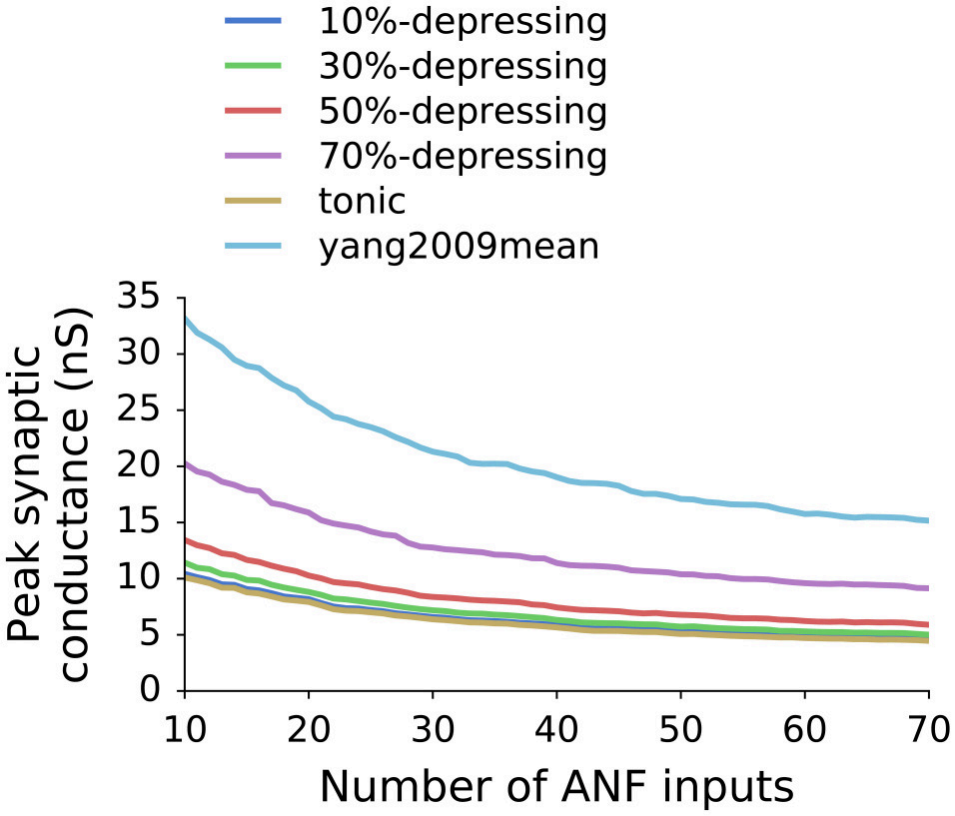
**Optimal peak synaptic conductance as a function of the number of ANF inputs to a GBC and the synapse type (depression level)**.

Figure 5 shows postsynaptic currents of two GBCs with 10%-depressing and yang2009mean synapses (Table 1) having optimal weights. The GBCs were stimulated with a 50 ms pure tone of 650 Hz. Each plot has a corresponding curve in Figure 4 with 35 ANF inputs. The optimal synaptic weights can be found in Figure 4 at the intersection of the target SR (dotted line, 7.5 spikes/s) and the corresponding curves. The optimal synaptic weights for all analyzed synapse types and number of inputs are plotted in Figure 6.

### 2.4. Implementation details

The model of GBCs was implemented in the NEURON simulation environment (Carnevale and Hines, 2006) and can be freely accessed, studied, and improved at https://github.com/mrkrd/cochlear_nucleus (Rudnicki and Hemmert, 2017). Python programming language was used to manage the simulations and analyze the data. The model input was generated using the free *cochlea* Python library, which we made publicly available at Rudnicki and Hemmert (2014). Specifically, the library includes the inner ear model of Zilany et al. (2014).

## 3. Results

This study mainly aimed to investigate the influence of different synapse types (tonic, with single, and with double exponential recovery), depression levels (0–70%), and the number of ANF inputs (10–70) on the firing properties of bushy cells. We generated *in vivo*-like stimulation patterns and analyzed VS and EI of the responses. The reference data came primarily from *in vivo* experiments of Joris et al. (1994).

### 3.1. Influence of synaptic depression on synchronization and entrainment

In the first set of simulations, we examined how different synapse types and depression levels influence synchronization and entrainment for cells with different CFs. We kept the number of ANF inputs constant, so every GBC was connected to 40 high-SR fibers. The stimulus was a train of 100 ramped pure tones (50 ms, 60 dB_SPL_) at CF (Joris et al., 1994).

Consistently with previous studies (Rothman et al., 1993; Xu-Friedman and Regehr, 2005; Joris and Smith, 2008), we found that GBCs improved phase locking relative to ANFs in the low-frequency range up to approximately 1.5 kHz. Synchronization was similar for all synaptic models and depression levels, in good agreement with experimental data. However, there was a large difference between entrainment for synapses with different depression levels. The EI was unrealistically low for depression levels >50% between 400 and 800 Hz. The synapse with *in vitro*-like depression and double exponential recovery (Yang and Xu-Friedman, 2009) failed to drive GBCs above 400 spikes/s. Synapses with depression levels from 0 to 50% produced EI in the range of the reference data.

### 3.2. Influence of the number of inputs on synchronization and entrainment

Spirou et al. (2005) found a large variability (9–69) of ANF inputs converging onto the soma of individual GBCs. To examine the influence of the number of inputs on phase locking and entrainment, we analyzed different convergence patterns ranging from 20–50 inputs for a 10%-depressing synapse. Because “high-sync” neurons are of the main interest for this study, we focused on bushy cells with average and high numbers of inputs. **Figure 8** shows both the VS and EI for the simulated GBCs. We also included the original experimental data points (triangles and stars) from Joris et al. (1994) and simulated ANFs (dotted line) for reference. **Figure 8** shows that the number of inputs has a strong effect on entrainment. High EI values for CFs up to 700 Hz could be reached only with a large number (50) of converging ANFs; smaller numbers of inputs caused a degradation of the EI. The change of entrainment due to a variation in the number of ANF inputs reflects the variability of the experimental data. The entire range of the measured data points was covered merely using a simple variation of the number of ANF inputs. It is further quantified in Section 3.3 where all data points were fitted to models by changing the number of ANF inputs. Compared to entrainment, the number of inputs had a smaller effect on phase locking, with a decreased number of ANF inputs leading to only a slight decrease of the VS.

### 3.3. Fitting number of ANF inputs and synapse types to individual data points of EI

**Figure 7:**
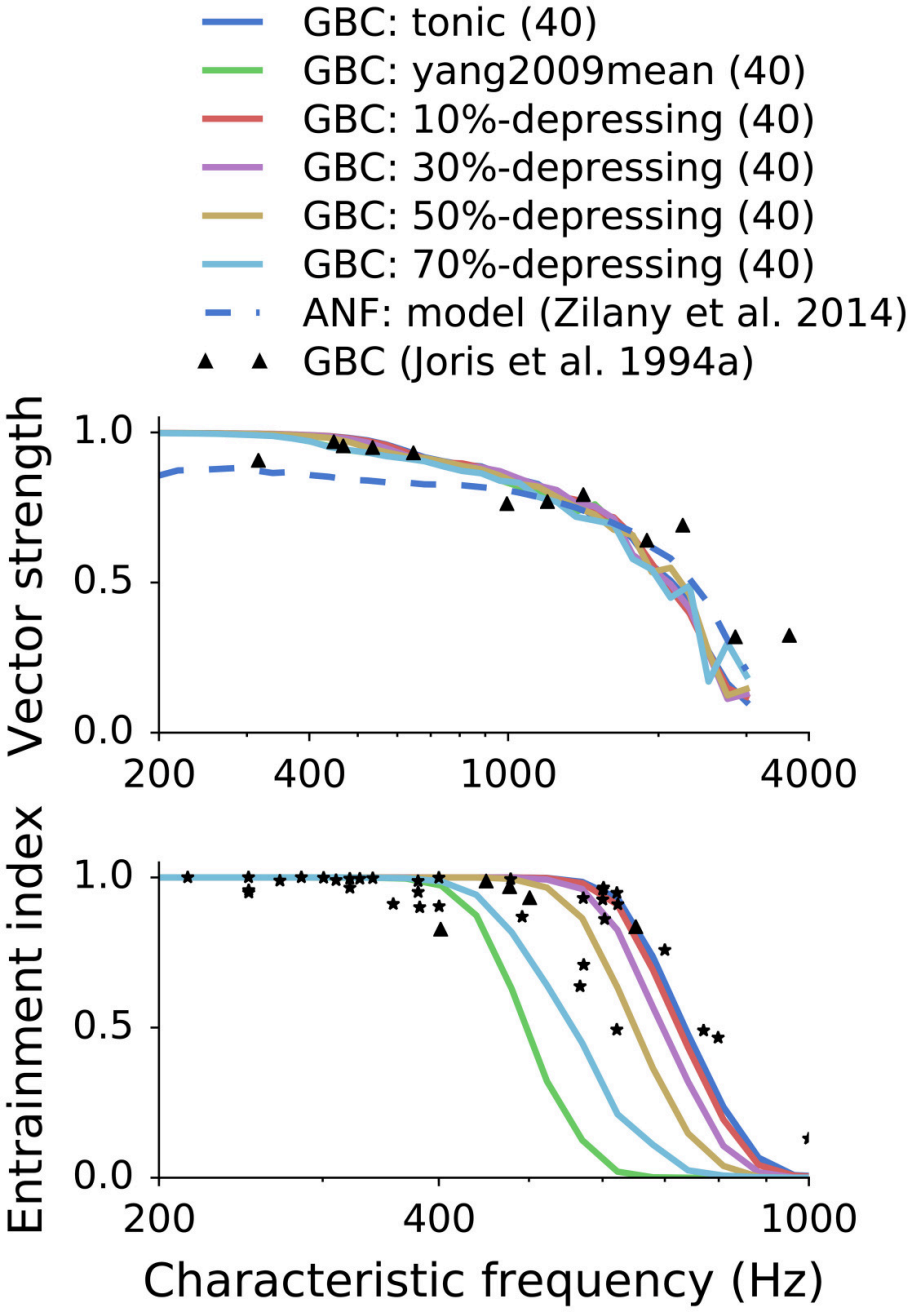
**Influence of synaptic depression on synchronization and entrainment**. The points in the plots represent experimental data digitized from Joris et al. (1994). Each GBC was excited by 40 high SR ANFs.

**Figure 8:**
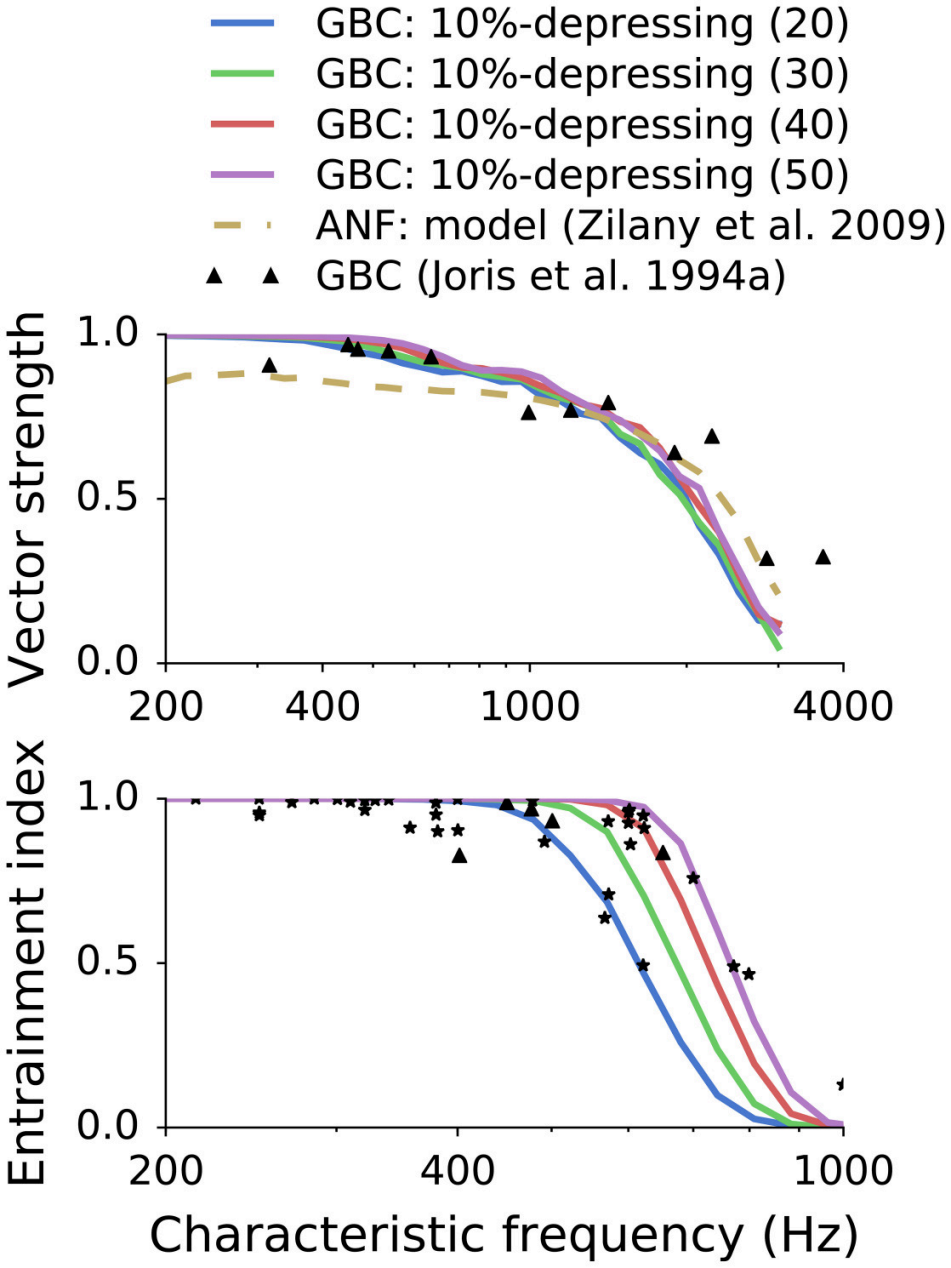
**Influence of the number of ANF inputs on synchronization and entrainment**. While the number of inputs only slightly influenced the VS, the effect on EI was strong. The variability of the experimental data can be explained in the model by the variation in the number of inputs to GBC. Reference data is from Joris et al. (1994).

**Figure 9:**
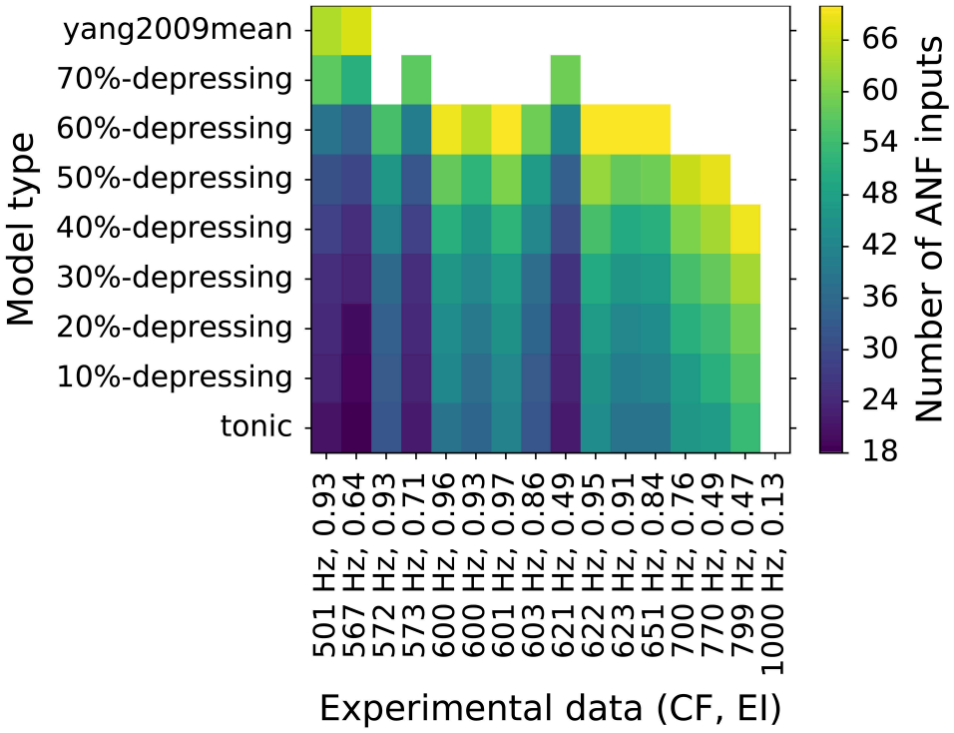
**Fitting of number of ANF inputs for every synapse type to EI data point from Joris et al. (1994)**. Only data points with CF > 500 Hz are shown for clarity. Colors encode the number of ANF inputs that were necessary to fit the data. Each data point is represented as a tuple (CF, EI) on the horizontal axis. The EI data points are also shown in the lower panels of Figures 7, 8. A white field means that no fit was possible for a given synapse type.

Previous simulations showed that VS does not change significantly for different synapse types (depression levels, Figure 7) or the number of ANF inputs (Figure 8). At the same time, EI strongly depends on both. To have better insight into the combined effect of the parameters, each synapse type was fitted to every experimental data point from the EI plot from Joris et al. (1994). The same data points are plotted in the lower panels of Figures 7, 8. The results of the fitting are shown in Figure 9 where colors encode the number of ANF inputs that was necessary to fit the data for each model. For clarity, only the frequencies in the transition region (CF > 500Hz), in which EI degrades, was plotted. Data in the low frequency region (CF <500Hz) could be reproduced by any synapse model given enough ANF inputs, which is also visible in Figures 7, 8. The possible number of ANF inputs varied from 10–70 as observed by Spirou et al. (2005). They also discovered that the majority of GBCs receive 23 or less inputs. This constraint holds only for a few fitted GBCs in the transition region and is possible for synapses with small depression levels (0–20%). Other fitted GBCs required more inputs. Fields with white color mean that it was not possible to fit the model to the given EI at the given CF. Models with depression >50% clould no longer reproduce data points with the highest CFs. The simulations also show that all (except one) EI data points could be reproduced by the less depressing models by varying the number of ANF inputs. SR of all modeled GBCs was set to 7.5 spikes/s. For completeness, Supplementary Material shows additional results for GBC models with higher SR.

## 4. Discussion

GBCs play an important role in the sound localization pathway. They are highly specialized for that function because of fast channel kinetics and fast synapses. Together with multiple inputs from ANFs, such specialized features enable them to enhance the precision of temporal coding. Our investigation showed how to optimally adjust synaptic weights for endbulbs of Held with different levels of depression to produce realistic GBC responses including “high-sync” neurons. Additionally, simulations showed that synapses with depression greater than 50% can reproduce less data points than synapses with smaller depression. The depression is likely to be weaker (0–20%), because strongly depressing synapses require much more ANF inputs. Synapses with exponential recovery mechanisms fitted to *in vitro* experiments with paired-pulse stimuli did not operate properly with *in vivo*-like stimuli in the most of the transition region (*CF* > 500Hz). It indicates that synaptic mechanisms which counteract vesicle depletion are activated *in-vivo*. It also shows that even though giant synapses have been studied in great detail before, it is still important to examine them as a part of a larger system, what provides further constrains. Finally, variation of the number of ANF inputs and synaptic depression can reproduce variability of entrainment observed *in vivo*. Last but not least, our modeled GBCs can generate realistic spike trains using a code that is freely available at https://github.com/mrkrd/cochlear_nucleus (Rudnicki and Hemmert, 2017) for researchers who are interested in investigating neuronal processing at higher stages of the auditory pathway, especially in sound localization (Encke and Hemmert, 2015).

### 4.1. Adjustment of synaptic weights

Our model consisted of three main components: ANF inputs, endbulbs of Held and a GBC soma. The properties of ANF inputs and GBC soma had been well studied before. We used accurate biophysical models with fixed parameters from Zilany et al. (2014) and Rothman and Manis (2003c). The inner ear model of Zilany et al. (2014) allowed us to generate realistic *in vivo*-like ANF spike trains. GBC soma, despite being relatively realistic, still lacked features, such as multiple compartments and inhibitory inputs. In particular, inhibitory inputs might play an important role in GBCs as they do in spherical bushy cells (Nerlich et al., 2014; Keine and Rübsamen, 2015; Kuenzel et al., 2015). Since we wanted to examine a variety of endbulbs of Held, we decided to use phenomenological synapse models that allowed us to easily modify model parameters in a controllable way. Thus we could examine how synaptic properties influence input-output characteristic of the system, but did not examine the exact mechanisms of depression.

Synaptic weights were fitted separately for each combination of synapse type and the number of synapses (ANF inputs). The main objective was to achieve large VS and EI while keeping the SR realistic. While the SR of GBCs can vary a lot (Spirou et al., 1990; Smith et al., 1993; Rhode, 2008) observed correlation between SR and CF. They noticed that GBCs at low CFs tend to have low SR and GBCs at high CFs have higher spontaneous activity. Because our model was adjusted for GBCs with low CFs it might not be valid for GBCs with higher CFs and SRs. Additionally, Kopp-Scheinpflug et al. (2008) showed indirectly similar pattern, where principal neurons in MNTB with low CF tend to have lower SRs than neurons with high CFs for various species. Firing rates of MNTB neurons are related to GBCs, because they are directly driven by GBCs through calyxes of Held which work as relays (Borst and Soria van Hoeve, 2012). However, the SRs of MNTB neurons were higher than SRs of GBCs.

Because the VS and EI (as well as driven rate) are a non-decreasing function of synaptic weights (Figure 4), the SR was the limiting factor during the parameter scan. The SR was set to 7.5 spikes/s which was the average SR of GBCs with CF <6 kHz from Spirou et al. (1990). The optimal synaptic weights for various numbers of ANF inputs and levels of synaptic depression are shown in Figure 6. The plot shows that the weights decrease with increasing number of ANF inputs to the asymptote of 0. Additionally, synapses with stronger depression have larger synaptic weights. The weights in the plot represent the initial synaptic conductance which is reduced during synaptic operation.

Rothman et al. (1993) presented a study of a bushy cell model with tonic synapses that was strongly influential for the design of our model. The authors performed a detailed parameter space scan varying the number of inputs and synaptic strengths. Their model could reproduce all typical peristimulus time histograms of bushy cells (PL, PL_N_, On) and improve synchronization. The main differences with our study were lower number of ANF inputs (1–20) and consideration of tonic synapses only. The model of Rothman et al. (1993) most successfully captured the properties of spherical bushy cells, whereas our model focused on GBCs. Furthermore, Kuenzel et al. (2015) and Nerlich et al. (2014) showed advancements in modeling of spherical bushy cells which included inhibition and dynamic synapses to better explain *in vitro* and *in vivo* experiments.

Spirou et al. (2005) presented a detailed biophysical model of GBCs. They combined structural data from electron microscopy with physiological data about vesicle release probability, synaptic depression, and receptor kinetics from Graham (2001). The model was tuned to closely reflect physiological data and successfully reproduced peristimulus time histograms of various GBCs. However, this model failed to improve the temporal precision for depressing synapses, with none of the simulated cells achieving a synchronization index larger than 0.9. In contrast, improved phase locking was one of the main goals of our model. We also examined various depression levels, but only for phenomenological synapse models.

### 4.2. Levels of depression in *in vivo*-like simulations

We presented three different types of models of endbulb of Held: tonic, with single, and with double exponential recovery synapse. Tonic synapses were reported in some earlier *in vivo* studies of endbulbs (Young and Sachs, 2008; Borst, 2010; Kuenzel et al., 2011) and calyxes (Lorteije et al., 2009) of Held. They also represent a boundary case when analyzing depression, because they are equivalent to 0%-depressing synapses. Simple depressing synapses with single exponential recovery allowed us to study a whole range of depression levels (0–70%). Finally, depressing synapse with double exponential recovery represented an average synapse from *in vitro* experiments, where the data was fitted to a double exponential function, see Figure 3 (Yang and Xu-Friedman, 2009). All synapse models were purely phenomenological, i.e., they did not attempt to explain the mechanism of depression. It allowed us to easily manipulate synapse parameters and observe the influence of the depression on firing properties in *in vivo*-like stimulations.

A qualitative comparison of two synapses is shown in Figure 5. It shows synaptic currents of a weakly depressing (10%-depressing) and a strongly depressing (yang2009mean, Table 1) synapse during spontaneous and driven activity. First, the responses during the spontaneous activity look very similar. This is a direct result of the optimization where all synapses were fitted to have a specific SR. Second, the average amplitude of the synaptic currents is smaller compared to the 10%-depressing synapse. The peaks often do not reach the threshold necessary to generate a single action potential. As a result, there are many failures in generating action potentials. The strong difference in the driven region between both synapses is expected, because they represent the two extreme cases: one of the least and one of the most depressing synapses in our study. The other depression levels are expected to lie between them and are shown next.

Figure 7 shows how different synapse types and levels of depression influence firing properties of GBCs in *in vivo*-like simulations. Interestingly, the depression level had little effect on phase locking. All GBC spike trains had VS greater than VS of ANF trains for frequencies <1 kHz, which is also observed *in vivo*. The results quantify how EI degrades with increasing synaptic depression. It is consistent with Figure 5 where the strongly depressing synapse was not able to drive GBCs to high firing rates. For the given number of ANF inputs (40), the maximum depression was approximately 50–60%. Stronger depression levels caused EI to be lower than the EI of the reference data. The interplay between the depression level and the number of ANF inputs is explained in the next paragraph.

Figure 9 shows fitting results of every depression model to individual EI data points in the transition region (*CF* <500 *Hz*) from Joris et al. (1994). The allowed number of ANF inputs was in the range of 10–70 (Spirou et al., 2005). If no fit was found, the corresponding field was left blank. Additional results including GBCs with higher SRs (20 spikes/s and 50 spikes/s) are shown in the Supplementary Material. First of all, in the low frequency region (not shown in Figure 9, but visible in Figure 7) all data could be reproduced by all examined synapses. It is consistent with *in vitro* observations of a large variability of the depressing synapses Yang and Xu-Friedman (2009). However, the double exponential recovery synapse, that was tuned explicitly to *in vitro* data, did not reach the desired entrainment for GBCs with *CFs* > 500 Hz, which could have two explanations:

1. The synaptic properties depend on the stimulus, e.g., a synapse might have different recovery time constants for paired-pulse (often used in slices) and ongoing (*in vivo*-like) stimuli as observed by Yang and Xu-Friedman (2015).
2. Physiological conditions *in vitro* and *in vivo* are responsible for different synaptic properties as suggested by Lorteije et al. (2009).

Therefore, for the *in vitro* synapse model to properly operate in *in vivo* conditions, requires either (a) adjustment of the synaptic parameters for the *in vivo* condition, or (b) a development of a more complex model (including the actual bio-physiological processes). This result also suggests that slice measurements of synaptic parameters cannot be easily adopted to *in vivo* conditions.

The finding above is consistent with many *in vivo* observations of calyx of Held, where there was a small depression observed (Wang et al., 2013, 2015; Di Guilmi et al., 2014) or no evidence of synaptic depression (Lorteije et al., 2009). Also, Kuenzel et al. (2011) and Young and Sachs (2008) did not observe evidence of strong synaptic depression *in vivo* in endbulbs of Held. Hermann et al. (2009) observed reduction (but not abolition) of the synaptic depression *in vitro* using conditioning with spontaneous activity. They concluded that calyxes of Held operate in chronic synaptic depression *in vivo*. A similar explanation came from Friauf et al. (2015) where they proposed that *in vivo* synapses are in their normal stimulation range and the *in vitro* synapses are in a “manic” state due to lack of the spontaneous stimulation. Additionally, a possibly similar effect *in vivo* was shown by Di Guilmi et al. (2014) and Wang et al. (2015) where calyxes associated with low-SR (<20 Hz) MNTB neurons had larger synaptic depression (23%) than high-SR neurons (8%). The difference between *in vivo* and *in vitro* synapses could be also partially explained by adjusting the *Ca*^2+^ concentration. Yang and Xu-Friedman (2015) showed large changes in synaptic depression by varying *Ca*^2+^ concentration from range 1 mmoll^−1^ to 2 mmoll^−2^ which resulted in depression varying from 0 to > 50%. Lorteije et al. (2009) observed reduction of the synaptic depression *in vitro* when varying *Ca*^2+^ concentration. They also noted that deviations in *Ca*^2+^ concentration was not enough to explain the difference between *in vitro* and *in vivo* synapses. Additionally, Yang and Xu-Friedman (2015) showed that not only the depression levels, but also time constant are different between *in vitro* and *in vivo* synapses, which suggest a different mode of operation *in vitro* and *in vivo*.

### 4.3. Number of ANF inputs

In the simulations, we varied the number of ANF inputs (10–70) similarly to the observations of Spirou et al. (2005). Figure 8 show that on the one hand, the number of ANF inputs did not significantly influence VS in pure tone stimulations. On the other hand, EI was strongly influenced by the number of ANF inputs. Figure 9 shows most EI data points can be reproduced by varying the number of ANF inputs for synapses with depression smaller than 50%. In addition, Spirou et al. (2005) observed that the majority of GBC received less than 23 inputs, which could be reached only by some synapses in the transition region (*CF* > 500 Hz) with low synaptic depression (0–20%).

### 4.4. Physiological consequences

The main finding of our study that the combination of strongly adapting inputs from ANFs with a biophysically plausible model of GBCs generates realistic output spike trains only when the endbulb of Held synapses show low depression levels has also important consequences on how this synapse works.

The synaptic terminals of the primary ANFs are remote from the soma. This distance limits and delays the delivery of new supplies to the synapse. For sustained operation terminals have to locally recycle their vesicles and neurotransmitter. GBCs are very specialized neurons, they can fire series of sustained and precisely phase-locked action potentials up to 700 Hz, despite decreasing (adapting) input from ANFs. The modeling results presented here show that this is only possible, if the endbulb of Held synapse has active mechanisms in place, which counteract depression. One mechanism is probably the high number of release sites (up to 150, Oleskevich et al., 2000; Nicol and Walmsley, 2002; Borst, 2010), in combination with a release probability, which has to be much smaller than the value of 0.6 determined *in vitro*. Only with low release probabilities vesicles can be spared such that the available vesicle pool size does not deplete too fast and too much during phases with high-frequency trains of presynaptic action potentials. Nevertheless, even small amount of vesicle depletion leads to depression and therefore compensating mechanisms have to be in place. Neher and T. Sakaba (2008) discuss that global intraterminal Ca^2+^ concentration, which increases during phases of high activity, accelerates vesicle recruitment. A second mechanism was described by Hosoi et al. (2007) in the calyx of Held, where they concluded that during a 100 Hz stimulus train the fusion probability increased during the initial 5–10 APs by a factor of five. If both factors compensate pool depletion, synaptic depression can be more or less suppressed such that GBCs can fire fast sequences of action potentials with little fatigue. For GBCs, this behavior is essential to shape and relay sustained and precisely phase-locked high-frequency pulse trains, which are required to evaluate interaural time and level differences in the lateral and medial superior olives.

## Author contributions

MR and WH are listed as authors according to the guidelines of Frontiers in Computational Neuroscience.

## Funding

This work was supported by the German Research Foundation (DFG) within the Priority Program “Ultrafast and temporally precise information processing: normal and dysfunctional hearing” SPP 1608 (HE6713), the Technische Universität München within the funding programme Open Access Publishing, and the German Federal Ministry of Education and Research within the Munich Bernstein Center for Computational Neuroscience (reference number 01GQ1004B).

### Conflict of interest statement

The authors declare that the research was conducted in the absence of any commercial or financial relationships that could be construed as a potential conflict of interest.

## Abbreviations

ANF: Auditory nerve fiber
CF: Characteristic frequency
EI: Entrainment index
GBC: Globular bushy cell
SR: Spontaneous rate
VS: Vector strength.

## References

[B1] Borst J. G. G. (2010). The low synaptic release probability *in vivo*. Trends Neurosci. 33, 259–266. 10.1016/j.tins.2010.03.003 20371122

[B2] Borst J. G. G. Soria van Hoeve J. (2012). The calyx of Held synapse: from model synapse to auditory relay. Ann. Rev. Physiol. 74, 199–224. 10.1146/annurev-physiol-020911-153236 22035348

[B3] Cao X.-J. Oertel D. (2010). Auditory nerve fibers excite targets through synapses that vary in convergence, strength, and short-term plasticity. J. Neurophysiol. 104, 2308–2320. 10.1152/jn.00451.2010 20739600 PMC3350034

[B4] Carnevale N. T. Hines M. L. (2006). The NEURON Book. Cambridge, UK: Cambridge University Press 10.1017/cbo9780511541612

[B5] Cook D. L. Schwindt P. C. Grande L. A. Spain W. J. (2003). Synaptic depression in the localization of sound. Nature 421, 66–70. 10.1038/nature01248 12511955

[B6] Di Guilmi M. N. Wang T. Inchauspe C. G. Forsythe I. D. Ferrari M. D. van den Maagdenberg A. M. J. M.. (2014). Synaptic gain-of- function effects of mutant Cav2.1 channels in a mouse model of familial hemiplegic migraine are due to increased basal [Ca2+]i. J. Neurosci. 34, 7047–7058. 10.1523/JNEUROSCI.2526-13.2014 24849341 PMC4028489

[B7] Encke J. Hemmert W. (2015). The relative timing of inhibitory and excitatory currents tunes the shift of best ITD in a detailed mammalian MSO network model, in The Auditory Model Workshop (Oldenburg).

[B8] Friauf E. Fischer A. U. Fuhr M. F. (2015). Synaptic plasticity in the auditory system: a review. Cell Tissue Res. 361, 177–213. 10.1007/s00441-015-2176-x 25896885

[B9] Xu-Friedman M. A. Regehr W. G. (2005). Dynamic-clamp analysis of the effects of convergence on spike timing. I. Many synaptic inputs. J. Neurophysiol. 94, 2512–2525. 10.1152/jn.01307.2004 16160092

[B10] Graham B. (2001). A computational model of synaptic transmission at the calyx of Held. Neurocomputing 38, 37–42. 10.1016/S0925-2312(01)00476-3

[B11] Hermann J. Grothe B. Klug A. (2009). Modeling short-term synaptic plasticity at the calyx of held using *in vivo*-like stimulation patterns. J. Neurophysiol. 101, 20–30. 10.1152/jn.90243.2008 18971300

[B12] Hosoi N. Sakaba T. Neher E. (2007). Quantitative analysis of calcium-dependent vesicle recruitment and its functional role at the calyx of held synapse. J. Neurosci. 27, 14286–14298. 10.1523/JNEUROSCI.4122-07.2007 18160636 PMC6673456

[B13] Joris P. X. Carney L. H. Smith P. H. Yin T. C. (1994). Enhancement of neural synchronization in the anteroventral cochlear nucleus. I. Responses to tones at the characteristic frequency. J. Neurophysiol. 71, 1022–1036. 8201399 10.1152/jn.1994.71.3.1022

[B14] Joris P. X. Smith P. H. (2008). The volley theory and the spherical cell puzzle. Neuroscience 154, 65–76. 10.1016/j.neuroscience.2008.03.002 18424004 PMC2486254

[B15] Keine C. Rübsamen R. (2015). Inhibition shapes acoustic responsiveness in spherical bushy cells. J. Neurosci. 35, 8579–8592. 10.1523/JNEUROSCI.0133-15.2015 26041924 PMC6605330

[B16] Kopp-Scheinpflug C. Tolnai S. Malmierca M. Rübsamen R. (2008). The medial nucleus of the trapezoid body: comparative physiology. Neuroscience 154, 160–170. 10.1016/j.neuroscience.2008.01.088 18436383

[B17] Kuenzel T. Borst J. G. G. van der Heijden M. (2011). Factors controlling the input-output relationship of spherical bushy cells in the gerbil cochlear nucleus. J. Neurosci. 31, 4260–4273. 10.1523/JNEUROSCI.5433-10.2011 21411667 PMC6623538

[B18] Kuenzel T. Nerlich J. Wagner H. Rübsamen R. Milenkovic I. (2015). Inhibitory properties underlying non-monotonic input-output relationship in low-frequency spher-ical bushy neurons of the gerbil. Front. Neural Circuits 9:14. 10.3389/fncir.2015.00014 25873864 PMC4379913

[B19] Liberman M. C. (1991). Central projections of auditory-nerve fibers of differing spontaneous rate. I. Anteroventral cochlear nucleus. J. Comp. Neurol. 313, 240–258. 10.1002/cne.903130205 1722487

[B20] Lorteije J. A. M. Rusu S. I. Kushmerick C. Borst J. G. G. (2009). Reliability and precision of the mouse calyx of held synapse. J. Neurosci. 29, 13770–13784. 10.1523/JNEUROSCI.3285-09.2009 19889989 PMC6666705

[B21] Neher E. Sakaba T. (2008). Multiple roles of calcium ions in the regulation of neurotransmitter release. Neuron 59, 861–872. 10.1016/j.neuron.2008.08.019 18817727

[B22] Nerlich J. Kuenzel T. Keine C. Korenic A. Rübsamen R. Milenkovic I. (2014). Dynamic fidelity control to the central auditory system: synergistic glycine/GABAergic inhibition in the cochlear nucleus. J. Neurosci. 34, 11604–11620. 10.1016/j.neuron.2008.08.019 25164657 PMC6608417

[B23] Nicol M. J. Walmsley B. (2002). Ultrastructural basis of synaptic transmission between endbulbs of Held and bushy cells in the rat cochlear nucleus. J. Physiol. 539(Pt 3), 713–723. 10.1113/jphysiol.2001.012972 11897843 PMC2290185

[B24] Oleskevich S. Clements J. Walmsley B. (2000). Release probability modulates short-term plasticity at a rat giant terminal. J. Physiol. 524, 513–523. 10.1111/j.1469-7793.2000.00513.x 10766930 PMC2269875

[B25] Osen K. K. (1969). Cytoarchitecture of the cochlear nuclei in the cat. J. Comp. Neurol. 136, 453–483. 10.1002/cne.901360407 5801446

[B26] Recio A. (2000). Representation of vowel stimuli in the ventral cochlear nucleus of the chinchilla. Hear. Res. 146, 167–184. 10.1016/S0378-5955(00)00111-8 10913893

[B27] Rhode W. S. (2008). Response patterns to sound associated with labeled globular/bushy cells in cat. Neuroscience 154, 87–98. 10.1016/j.neuroscience.2008.03.013 18423882 PMC2518325

[B28] Rhode W. S. Greenberg S. (1994). Encoding of amplitude modulation in the cochlear nucleus of the cat. J. Neurophysiol. 71, 1797–1825. 8064349 10.1152/jn.1994.71.5.1797

[B29] Rhode W. S. Oertel D. Smith P. H. (1983). Physiological response properties of cells labeled intracellularly with horseradish peroxidase in cat ventral cochlear nucleus. J. Comp. Neurol. 213, 448–463. 10.1002/cne.902130408 6300200

[B30] Rothman J. S. Young E. D. Manis P. B. (1993). Convergence of auditory nerve fibers onto bushy cells in the ventral cochlear nucleus: implications of a computational model. J. Neurophysiol. 70, 2562–2583. 8120599 10.1152/jn.1993.70.6.2562

[B31] Rothman J. S. Manis P. B. (2003a). Differential expression of three distinct potassium currents in the ventral cochlear nucleus. J. Neurophysiol. 89, 3070–3082. 10.1152/jn.00125.2002 12783951

[B32] Rothman J. S. Manis P. B. (2003b). Kinetic analyses of three distinct potassium conductances in ventral cochlear nucleus neurons. J. Neurophysiol. 89, 3083–3096. 10.1152/jn.00126.2002 12783952

[B33] Rothman J. S. Manis P. B. (2003c). The roles potassium currents play in regulating the electrical activity of ventral cochlear nucleus neurons. J. Neurophysiol. 89, 3097–3113. 10.1152/jn.00127.2002 12783953

[B34] Rudnicki M. Hemmert W. (2014). Cochlea: Inner Ear Models in Python. 10.5281/zenodo.61462 Available online at: https://github.com/mrkrd/cochlea

[B35] Rudnicki M. Hemmert W. (2017). Cochlear_Nucleus: Computational Models of the Globular Bushy Cells in the Ventral Cochlear Nucleus (v1.4). Zenodo. 10.5281/zenodo.345413 Available online at: https://github.com/mrkrd/cochlear_nucleus

[B36] Smith P. H. Joris P. X. Yin T. C. T. (1993). Projections of physiologically characterized spherical bushy cell axons from the cochlear nucleus of the cat: evidence for delay lines to the medial superior olive. J. Comp. Neurol. 331, 245–260. 10.1002/cne.903310208 8509501

[B37] Spirou G. A. Brownell W. E. Zidanic M. (1990). Recordings from cat trapezoid body and HRP labeling of globular bushy cell axons. J. Neurophysiol. 63, 1169–1190. 2358868 10.1152/jn.1990.63.5.1169

[B38] Spirou G. A. Rager J. Manis P. B. (2005). Convergence of auditory-nerve fiber projections onto globular bushy cells. Neuroscience 136, 843–863. 10.1016/j.neuroscience.2005.08.068 16344156

[B39] Taschenberger H. Woehler A. Neher E. (2016). Superpriming of synaptic vesicles as a common basis for intersynapse variability and modulation of synaptic strength. Proc. Natl. Acad. Sci. U.S.A. 113, E4548–E4557. 10.1073/pnas.1606383113 27432975 PMC4978258

[B40] Tsodyks M. V. Markram H. (1997). The neural code between neocortical pyramidal neurons depends on neurotransmitter release probability. Proc. Natl. Acad. Sci. U.S.A. 94, 719–723. 10.1073/pnas.94.2.719 9012851 PMC19580

[B41] Wang T. de Kok L. Willemsen R. Elgersma Y. Borst J. G. G. (2015). *In vivo* synaptic transmission and morphology in mouse models of Tuberous sclerosis, Fragile X syndrome, Neurofibromatosis type 1, and Costello syndrome. Front. Cell. Neurosci. 9:234. 10.3389/fncel.2015.00234 26190969 PMC4490249

[B42] Wang T. Rusu S. I. Hruskova B. Turecek R. Borst J. G. G. (2013). Modulation of synaptic depression of the calyx of Held synapse by GABAB receptors and spontaneous activity. J. Physiol. 591, 4877–4894. 10.1113/jphysiol.2013.256875 23940376 PMC3800460

[B43] Wang Y. Manis P. B. (2008). Short-term synaptic depression and recovery at the mature mammalian endbulb of held synapse in mice. J. Neurophysiol. 100, 1255–1264. 10.1152/jn.90715.2008 18632895 PMC2544465

[B44] Yang H. Xu-Friedman M. A. (2008). Relative roles of different mechanisms of depression at the mouse endbulb of held. J. Neurophysiol. 99, 2510–2521. 10.1152/jn.01293.2007 18367696 PMC2905879

[B45] Yang H. Xu-Friedman M. A. (2009). Impact of synaptic depression on spike timing at the endbulb of held. J. Neurophysiol. 102, 1699–1710. 10.1152/jn.00072.2009 19587324 PMC2894642

[B46] Yang H. Xu-Friedman M. A. (2015). Skipped-stimulus approach reveals that short-term plasticity dominates synaptic strength during ongoing activity. J. Neurosci. 35, 8297–8307. 10.1523/JNEUROSCI.4299-14.2015 26019343 PMC4444548

[B47] Young E. D. Sachs M. B. (2008). Auditory nerve inputs to cochlear nucleus neurons studied with cross-correlation. Neuroscience 154, 127–138. 10.1016/j.neuroscience.2008.01.036 18343587 PMC2478560

[B48] Zilany M. S. A. Bruce I. C. Carney L. H. (2014). Updated parameters and expanded simulation options for a model of the auditory periphery. J. Acoust. Soc. Am. 135, 283–286. 10.1121/1.4837815 24437768 PMC3985897

[B49] Zilany M. S. A. Bruce I. C. Nelson P. C. Carney L. H. (2009). A phenomenological model of the synapse between the inner hair cell and auditory nerve: long-term adaptation with power-law dynamics. J. Acoust. Soc. Am. 126, 2390–2412. 10.1121/1.3238250 19894822 PMC2787068

