# Supplementary material for: High Entrainment Constrains Synaptic Depression Levels of an In vivo Globular Bushy Cell Model
Source: Front Comput Neurosci. 2017 Mar 20;11:16. doi: 10.3389/fncom.2017.00016 (PMC5357671; doi:10.3389/fncom.2017.00016)
Supplement: Supplementary file 1 [file DataSheet1.PDF]

Supplementary Material

High entrainment constrains synaptic depression levels of an *in vivo* globular bushy cell model

Marek Rudnicki[1*] and Werner Hemmert[1]

[1]Bio-Inspired Information Processing, Technische Universität München, Germany

[*]Correspondence: marek.rudnicki@tum.de

GBCs with high spontaneous rates

Generally, globular bushy cells (GBC) can have various spontaneous rates (SR) (Rhode 2008). However, Spirou, Brownell, and Zidanic (1990) and Smith, Joris, and Yin (1993) observed correlation between SR and characteristic frequency (CF). They noticed that low-CF neurons tend to have low SR. This property was used throughout the paper, where we focused on low-CF (low-SR) neurons. Here, we would like to present results of GBC simulations with higher SRs: 20 spikes/s and 50 spikes/s.

Fig. 1 and Fig. 2 show fitting of each synaptic model in the study to individual entrainment index (EI) data points from Joris et al. (1994). GBC models in Fig. 1 had the SR of 20 spikes/s and 50 spikes/s in Fig. 2. Colors indicate the number of ANF inputs that was necessary to replicate a given EI at the given CF. White color indicates that no fit was possible. For clarity, only data from the transition region ($CF > 500\,\mathrm{Hz}$) is shown, because the data for lower CF could be reproduced by all models.

Results show that GBCs with higher SRs require require less synaptic inputs for a given synapse type to replicate each data point. Additionally, GBCs with *yang2009mean* synapses, modeled directly from *in vitro* experiments of Yang and Xu-Friedman (2009), could produce valid entrainment for some data points in the transition region. Finally, the data point at 1 kHz could be also replicated but only by synapses with limited depression and large number of ANF inputs at higher SRs.

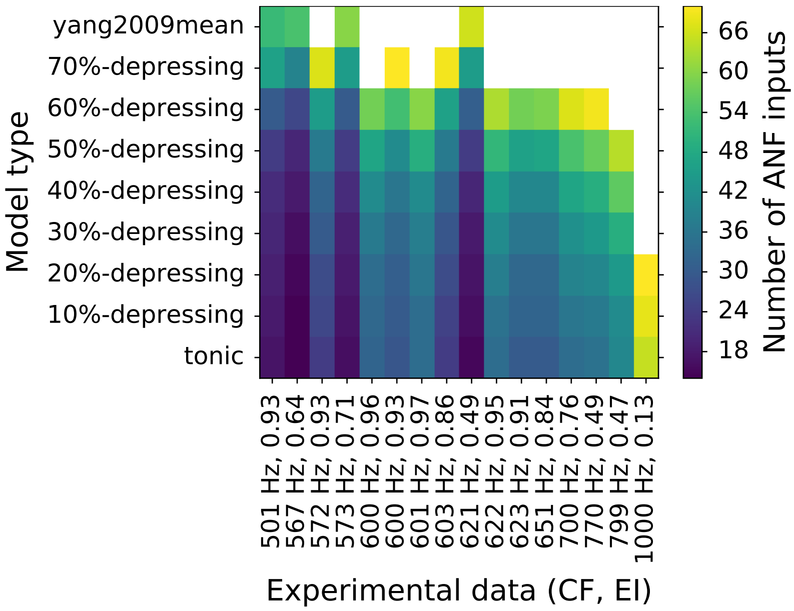

Figure 1: Fitting of the number of ANF inputs for every synapse type to individual EI data point from Joris et al. (1994). Only data points with CF $> 500\,\mathrm{Hz}$ are shown for clarity. Colors encode the number of ANF inputs that were necessary to fit the data. Each data point is represented as a tuple (CF, EI) on the horizontal axis. The EI data points are also shown in the main manuscript in Fig. 7 and Fig. 8. White fields mean that no fit was possible for a given synapse type. The SR of GBC models was set to 20 spikes/s.

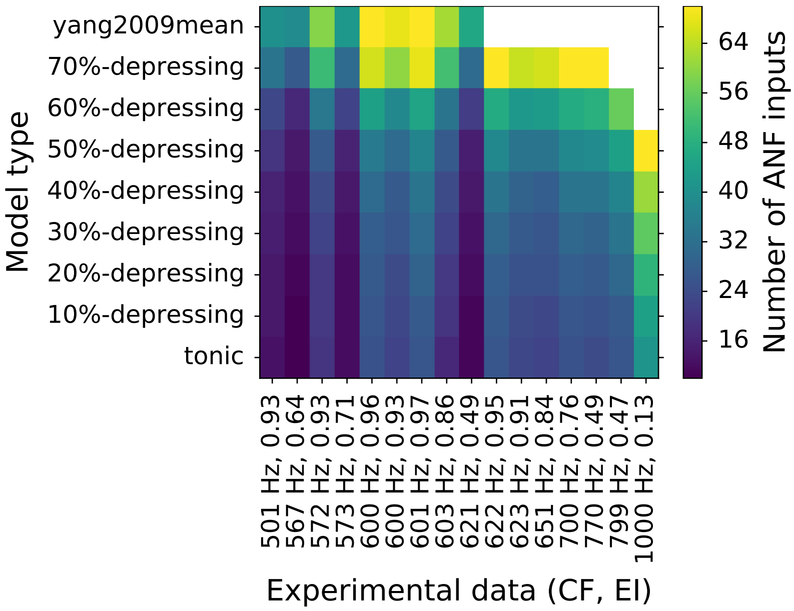

Figure 2: Fitting of the number of ANF inputs for every synapse type to individual EI data point from Joris et al. (1994). Only data points with CF > 500 Hz are shown for clarity. Colors encode the number of ANF inputs that were necessary to fit the data. Each data point is represented as a tuple (CF, EI) on the horizontal axis. The EI data points are also shown in the main manuscript in Fig. 7 and Fig. 8. White fields mean that no fit was possible for a given synapse type. The SR of GBC models was set to 50 spikes/s.

References

Joris, P. X., L. H. Carney, P. H. Smith, and T. C. Yin (1994). "Enhancement of neural synchronization in the anteroventral cochlear nucleus. I. Responses to tones at the characteristic frequency." In: *Journal of neurophysiology* 71.3, pp. 1022–1036. ISSN: 0022-3077.

Rhode, W. S. (2008). "Response patterns to sound associated with labeled globular/bushy cells in cat". In: *Neuroscience* 154.1, pp. 87–98. ISSN: 03064522. DOI: 10.1016/j.neuroscience.2008.03.013.

Smith, P. H., P. X. Joris, and T. C. T. Yin (1993). "Projections of physiologically characterized spherical bushy cell axons from the cochlear nucleus of the cat: Evidence for delay lines to the medial superior olive". In: *The Journal of Comparative Neurology* 331.2, pp. 245–260. ISSN: 1096-9861. DOI: 10.1002/cne.903310208.

Spirou, G. A., W. E. Brownell, and M. Zidanic (1990). "Recordings from cat trapezoid body and HRP labeling of globular bushy cell axons". In: *J Neurophysiol* 63.5, pp. 1169–1190.

Yang, H. and M. A. Xu-Friedman (2009). "Impact of synaptic depression on spike timing at the endbulb of held." In: *Journal of neurophysiology* 102.3, pp. 1699–1710. ISSN: 0022-3077. DOI: 10.1152/jn.00072.2009.
